# Evaluation of *Struthio camelus* eggshell as an in vitro alternative to extracted human teeth in preliminary screening studies on dental erosion

**DOI:** 10.1002/cre2.742

**Published:** 2023-04-27

**Authors:** Abubaker Qutieshat, Andrew Graham Mason, Richard Graham Chadwick

**Affiliations:** ^1^ Department of Adult Restorative Dentistry Oman Dental College Muscat Sultanate of Oman; ^2^ Department of Restorative Dentistry Dundee Dental Hospital & School Dundee UK; ^3^ School of Dentistry University of Dundee Dundee UK

**Keywords:** dental, eggshell, enamel, erosion

## Abstract

**Objectives:**

This in vitro work investigates the potential of ostrich eggshell as a substitute for extracted human teeth in preliminary screening studies on dental erosion. Additionally, it aims to demonstrate the potential of ostrich eggshell compared to human enamel in evaluating the efficacy of a preventive agent in protecting against dental erosion, using an artificial mouth model.

**Methods:**

The experiment utilized 96 erosion testing specimens from each substrate, human enamel, and ostrich eggshell. The specimens were subjected to six different experimental regimens of increasing erosive challenge, simulating the consumption of an acidic drink. The acidic drink was delivered at a consistent volume and duration range. Both artificially stimulated and unstimulated saliva flowed throughout the experimental regimens. Surface hardness was measured using a Through‐Indenter Viewing hardness tester with a Vickers diamond, while surface profiling was done using a surface contacting profilometer with a diamond stylus. An automated chemistry analyzer system was used to detect calcium and phosphate ions.

**Results:**

The study found that ostrich eggshell specimens demonstrated predictable surface loss, hardness drop, and ion loss due to the acidic challenge. Meanwhile, enamel appeared to fall short in terms of surface hardness predictability. The transient hardness loss phase, which manifests as an overlooked decrease in surface hardness despite significant ion and structural loss, may explain this phenomenon.

**Conclusions:**

The experiment showed that assessing surface loss is essential in addition to hardness testing, particularly as certain experimental conditions may produce a false perception of tissue recovery despite the actual surface loss. By analyzing the response of ostrich eggshell specimens to erosive challenges, researchers were able to identify an “overlooked” reduction in hardness in enamel specimens. The differences in the structure, chemical composition, and biological response to erosion in the presence of artificial saliva between enamel and ostrich eggshell could explain their distinct behaviors.

## 1 INTRODUCTION

A modern‐day definition of dental erosion, based on current perceptions, knowledge, and conceptions, defines it as a chemo‐mechanical phenomenon in which tooth surface structure is lost as a result of acidic challenge and mechanical pressure (Lussi & Carvalho, 2014). This distinguishes it from dental decay, which is caused by the fermentation of carbohydrates by bacteria found in dental plaque adhered to the tooth's surface.

Traditionally, laboratory research on dental erosion has utilized extracted human teeth to investigate etiological effects and preventive regimes. We have reported previously that the collection of such teeth is in decline (Qutieshat et al., 2020) and perhaps with the COVID pandemic the situation has worsened. Although any alternative substrate cannot fully replace a human tooth, it is our experience that an alternative be used in large‐scale testing,  as it would reserve scarce tissue for ultimate testing of, for example, promising preventive agents. This paper seeks to evaluate one such alternative (ostrich eggshell), comparing it to human tooth tissue. While the chemistries differ, it is important to keep an open mind about its potential utility.

The eggs of an ostrich are the largest of any avian species, measuring around 2 mm in thickness (ranging from 1.6 to 2.2 mm). Eggshells are a naturally occurring biocomposite made up of organic and inorganic components. More than 97% of an ostrich eggshell composition is comprised of inorganic mineral components, with calcium carbonate accounting for 97.4%, magnesium phosphate accounting for 1.9%, and tricalcium phosphate accounting for 0.7% (Yadao et al., 2004). It has been estimated that the ostrich eggshell has a calcium content of 370 mg/g and a phosphorus content of 0.2 mg/g (Szczerbińska & Wiercińska, 2010).

The egg's distinctive, dense structure confers exceptional mechanical qualities on it. It is capable of withstanding an applied force of more than 50 kg. Because the ostrich eggshell does not have a cuticle layer or any other shell accessory material, unlike the eggshells of other avian species, the topmost layer of the ostrich eggshell is a continuous unit that has a considerable thickness and consistent structure (>1.8 mm). Additionally, the vertical crystal layer has an amorphous crystalline structure devoid of porosity (Cooper, 2001; Cooper et al., 2009).

Furthermore, the eggshell's thickness, which can sometimes exceed 2 mm, makes it a promising substrate for various surface investigations in dentistry research. Ostrich eggshell is considered operator‐friendly from a laboratory standpoint since it is simple and convenient to cut to any shape with a dental rotary cutting instrument. Each ostrich egg produces approximately 300 g of eggshell. Additionally, the unique geometry of an eggshell allows it to withstand autoclaving without losing any of its biological properties (Yadao et al., 2004).

An erosion prevention agent that has often been evaluated by conventional means is fluoride. It gives a baseline against which to compare findings from the ostrich substrate utilized in this work. Fluoride has long been recognized as an efficient mineral preservation agent due to its capacity to build a protective shield on the surface of enamel composed mainly of calcium fluoride. This will prevent further enamel erosion and stimulate remineralization, and it will also add a mineralized barrier between the acid and the enamel below. As a result, regular brushing with fluoride toothpaste has been promoted to minimize enamel demineralization while simultaneously promoting its remineralization (Aykut‐Yetkiner et al., 2014).

Fluoride delivery vehicles other than toothpastes include gels, solutions, and varnishes. Each contains a variety of fluorinated chemicals, including sodium‐, stannous‐, ammonium‐, titanium‐, and acidulated phosphate‐fluoride (O'mullane et al., 2016). Literature suggests that these chemicals’ dual protective abilities—sticking to tooth structure via its resinous component and releasing fluoride ions onto the tooth structure's surroundings—help prevent mineral loss in response to acidic stimuli (Lins et al., 2021). High fluoride concentrations result in the creation of calcium fluoride, which acts as a physical barrier, allowing fluoride to exert its impact for a longer period of time.

The hypothesis tested in this study was that in ostrich eggshells, surface tissue loss would increase, and hardness would drop in response to an acidic beverage introduced to substrates via an artificial mouth model, and that such an effect is comparable to that of human enamel. The aims of this study were twofold; (Lussi & Carvalho, 2014) to investigate the suitability of ostrich eggshell as an alternative to extracted human teeth in preliminary screening studies on dental erosion (validation), and (Qutieshat et al., 2020) to demonstrate the potential utility of ostrich eggshell compared to human tooth tissue in evaluating the efficacy of a preventive agent in protecting from dental erosion (application).

## 2 METHODOLOGY

A novel testing environment housed 16 erosion‐testing substrate specimens that were subjected to six different experimental regimens of increasing erosive challenge, simulating the consumption of an acidic drink on separate occasions. Each set of specimens received only one of the experimental regimens (Table 1). The acidic drink was delivered in a standardized range of volumes and durations according to Qutieshat et al. (2015). Both stimulated and unstimulated artificial saliva flowed throughout the experimental regimens. An ethical approval to conduct this study was obtained from the NHS REC and NHS Tayside R&D.

**Table 1:** Summary of experimental regimens adopted in the validation and application sections of this work.

Regimen	Drink	Duration	Rest cycles	Number of erosive cycles	Erosive cycle dose	Total number of cans per specimen	Preventive measure	Code
Validation								
Short‐1 dose	Coca‐Cola	5 days	Days 1 and 5	3	1 can	3 cans	None	Regimen 1
Long‐ 1 dose	Coca‐Cola	7 days	Days 1 and 7	5	1 can	5 cans	None	Regimen 2
Short‐2 dose	Coca‐Cola	5 days	Days 1 and 5	3	2 can	6 cans	None	Regimen 3
Long‐ 2 dose	Coca‐Cola	7 days	Days 1 and 7	5	2 can	10 cans	None	Regimen 4
Application								
Short‐1 dose	Coca‐Cola	5 days	Days 1 and 5	3	1 can	3 cans	None	Unprotected
Short‐1 dose	Coca‐Cola	5 days	Days 1 and 5	3	1 can	3 cans	Fluor Protector S	Protected

Ostrich eggshell specimens were cut into 1 cm^2^ square‐shaped specimens using a diamond‐coated bur with constant cooling. The final thickness was 3 mm after glueing the 2‐mm specimens to the 1‐mm‐thick plexiglass cylinders that served as a base. Caries‐free extracted molars were chosen from the anonymous tooth collection of dental research teeth (The Conservation Collection registered with the Tayside Biorepository). The roots of each tooth were removed just above the cemento‐enamel junction, and the remaining tooth portion was embedded in acrylic resin in 3‐cm‐diameter cylindrical molds, vertically aligned. A slow‐speed diamond saw running at 450 rpm (Isomet Buehler Ltd.) was used to cut 3‐mm‐thick sagittal slices in a mesiodistal direction. Dentin‐free enamel‐only samples were chosen for experimental diets involving ion loss tracing. The specimens were kept in tap water until they were used. Once used, the teeth were disposed of anonymously.

**Figure 1:**
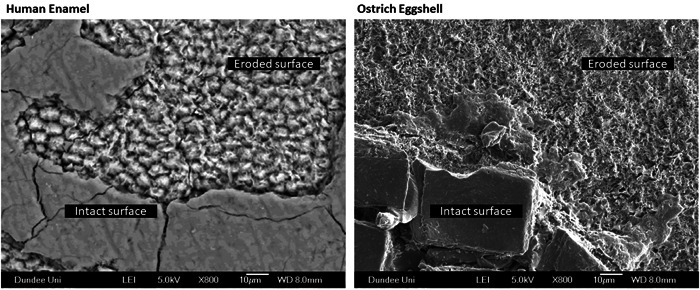
Scanning electron microscopy images displaying surface alterations of enamel (left) and ostrich eggshell (right) after exposure to 32% phosphoric acid, illustrating the comparable changes in response to the acidic challenge.

Before the main experiment, an exploratory pilot study was conducted with a scanning electron microscope (SEM) to better understand surface morphology changes in response to acidic challenges. Half of the exposed substrate surfaces were covered with acid‐resistant tape, and phosphoric acid (32% concentration) was applied for 30 s to the exposed substrate surfaces. As a result, enamel (Figure 1, left) and ostrich eggshell (Figure 1, right) underwent comparable surface alterations in response to phosphoric acid.

Baseline specimen surface microhardness and surface profile were determined before conducting any tests. Surface microhardness testing was performed using a Through‐Indenter Viewing (TIV) hardness tester to quantitatively and qualitatively measure the surface hardness of the specimens (GE Inspection TechnologiesK). With the TIV device, hardness was measured using Vickers indentations at a 1000 g load. Vickers diamond penetration is captured by a CCD camera embedded in the probe. All specimens were subjected to 10 baseline measurements of surface hardness, which were used to compute the percentage loss of surface hardness during each diet. The following formula was used to compute the relative hardness percentage of each specimen: hardness percentage = (hardness value)/(mean baseline hardness) × 100.

The ostrich eggshell surface loss was measured using a profilometer (Planer SF220 Surface Profilometer; Planer Products Ltd.) with a diamond stylus moving across the specimen surface with the help of markings made on the plexiglass base surface with an acid‐resistant permanent marker that included reference points for positioning and guidance lines for movement in a straight line. The markings allowed for the precise and repeatable positioning of specimens during profilometry measurements.

The surface profilometer device employed a diamond stylus with a tip diameter of 20 µm that moved in a straight line at a rate of 10 mm/min. The difference between the baseline/reference area and the average value over the length of the trace was used to calculate enamel/eggshell surface loss. The instrument's software determined the mean height loss across each profile.

Before the experiment, all specimens were exposed to three baseline surface profilometry readings that served as reference points for calculating any surface loss. Following the experiment, three additional readings were taken, and the mean value of the surface loss depth relative to baseline values was calculated.

Artificial saliva‐stimulated and unstimulated variants were prepared in‐house using the formulations described by Qutieshat et al. (2018). To detect any traces of calcium and phosphate ions in the drink/saliva mixture solution, an automated chemistry analyzer system was used (ADVIA® 2400; Siemens). To conduct daily analyses of the saliva/drink mixture solution, three 10‐mL universal vials were filled with the solution and kept at 4°C until all of the experiments were completed. The vials were then subjected to a chemical analysis, from which an average ion loss value was derived. The existing ion concentrations in the artificial saliva/test beverage solution were deducted.

### 2.1 Part 1: Validation—Human enamel versus ostrich eggshell

The effects of a variety of drinking behavior parameters on human enamel and *Struthio camelus* eggshell were examined in this article. This included the evaluation of both the volume of consumption and the duration of consumption on a daily basis. A portion of the methodology was previously discussed in a publication by our group (Qutieshat et al., 2018). The erosion cycles, dubbed regimens, were administered to specimens via an artificial mouth model constructed by our study group based on human physiological values (Qutieshat et al., 2015, 2018). A regimen is defined in this work as an experimental run that consists of a number of cycles during which specimens are subjected to artificial saliva and a test drink. Regimens lasted 5 or 7 days. The model operated continuously throughout each day of operation and consisted of three daily periods: daytime “waking hours,” nighttime “sleeping hours,” and an offensive (stimulated) phase. At physiological rates, artificial unstimulated saliva flowed through the system during the day and night, while stimulated artificial saliva flowed at increased rates in conjunction with the test drink (Coca‐Cola Regular; Coca‐Cola Enterprises Ltd.; pH = 2.47) during the stimulated period. This part tested four distinct regimens, which are presented in Table 1. Ion tracing was conducted only for the validation part of this work.

### 2.2 Part 2: Application—The effect of Fluor Protector

A 5‐day regimen comprising of three erosive cycles was conducted (short duration regimen and single‐dose drink). Fluor Protector S (Ivoclar Vivadent) was used to cover each of the eight enamel, and ostrich eggshell specimens were utilized in this study according to the manufacturer's instructions. The varnish was applied directly to the specimen surfaces in thin layers using the included brush in the package (Vivabrush G; Ivoclar Vivadent). The varnish was given 1 min to dry before the samples were incubated at 37°C for 60 min.

The erosive cycles began on the second and ended on the fourth, allowing for two erosion‐free days, one at the beginning and the other at the end of each regimen. Each erosive cycle included the consumption of one 330‐mL can of the test drink over a 22‐min period. In this work, three cans were consumed by each specimen. Surface hardness and tissue loss were determined on specimens twice: before the regimen's initiation and following its termination. The two regimens studied in this section are summarized in Table 1.

## 3 RESULTS

### 3.1 Part 1: Validation—Human enamel versus ostrich eggshell

#### 3.1.1 Surface hardness

**Figure 2:**
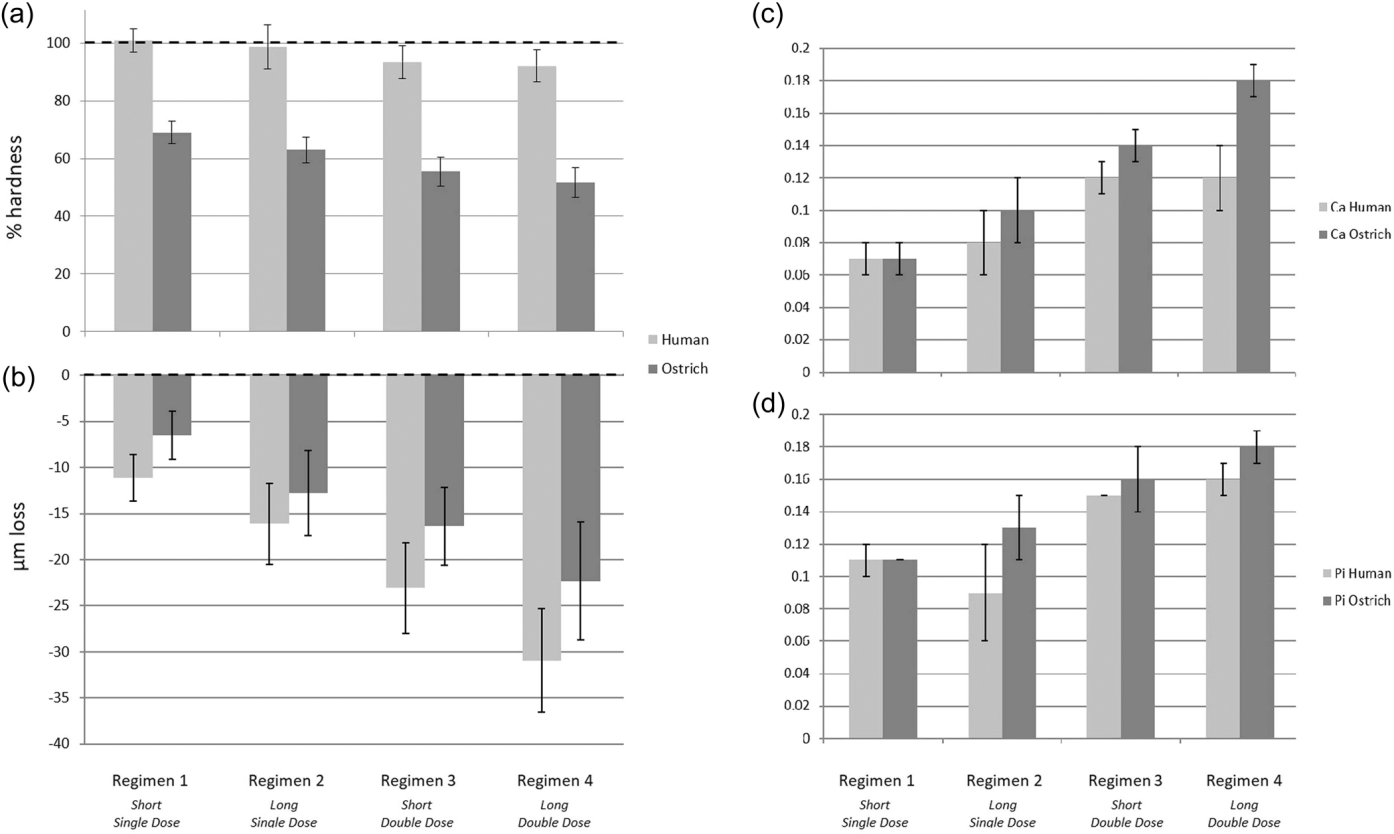
(a) Surface hardness as a percentage of preregimen values (100% hardness) for both substrates (mean ± SD). (b) Surface loss in µm (mean ± SD) over two runs for both substrates relative to preregimen values (0.00 µm). Nota bene: A positive loss value indicates material loss. (c) Calcium ion loss in mM/day for both substrates (mean ± SD). (d) Phosphate ion loss in mM/day for both substrates (mean ± SD).

Figure 2a depicts a plot of surface hardness change versus baseline values (100% relative hardness). The plot demonstrates that the ostrich eggshell hardness percentage values were much lower than enamel for all regimens. One‐way analysis of variance (ANOVA) analysis demonstrated that substrates had a highly significant effect on regimens 1, 2, 3, and 4 (*p* = .0001). Despite this, there was a uniform decrease in surface hardness across all test samples. None of the TIV images demonstrated any surface damage arising from profilometry.

#### 3.1.2 Surface loss

Figure 2b shows a plot of surface loss versus preregimen profilometry baseline values. The plot indicates that all treatments resulted in a marginally smaller surface loss from ostrich eggshell than from enamel. One‐way ANOVA analysis found that specimen type had no influence on regimens 1 and 2, but did have a significant effect on regimen 3 (*p* < .05) and a very highly significant effect on regimens 3 and 4 (*p* = .0001). It appears that the general trend of surface loss is the same across tissues, even though regimens 3 and 4 showed a statistically significant difference.

#### 3.1.3 Ion loss

Figures 2c and 1d show plots of calcium and phosphate ion loss. The plots demonstrate that daily ion loss values for ostrich eggshells were very similar to those for human enamel across all but the most aggressive regimen (i.e., regimen 4). On regimen 4, calcium loss from ostrich eggshells was significantly lower and phosphate loss was significantly higher than in enamel. One‐way ANOVA analysis revealed that the substrate had no effect on the amount of phosphate ion loss in any of the regimens (*p* > .05), but had a very significant effect on calcium loss in regimen 4 only (*p* < .001). The remaining differences were not statistically significant (*p* > .05).

### 3.2 Part 2: Application—The effect of Fluor Protector

#### 3.2.1 Human enamel

##### Surface hardness

**Figure 3:**
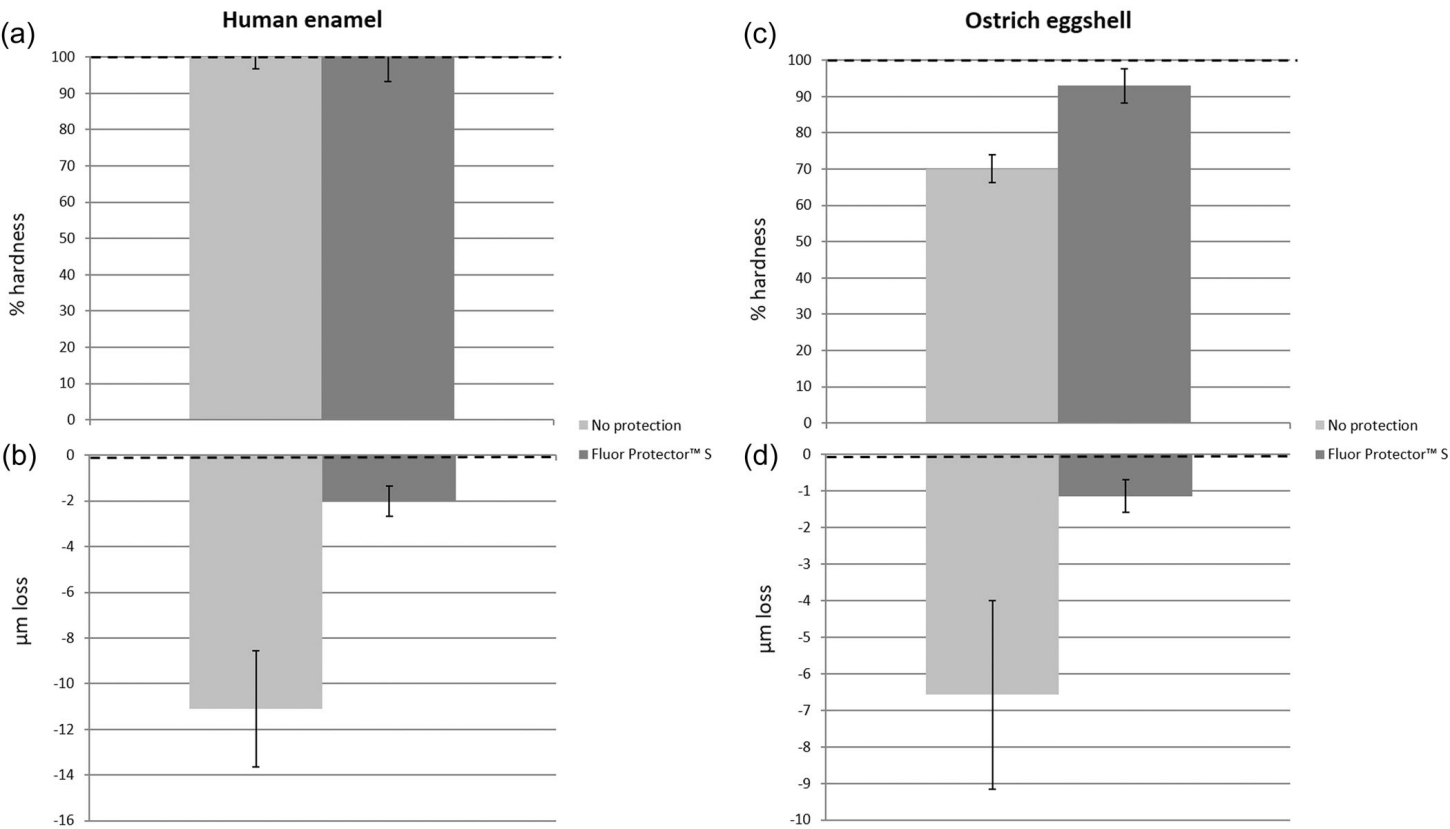
(a) Surface hardness of human enamel as a percentage of preregimen values (mean ± SD) (100% hardness). (b) Values for human enamel surface loss in micrometer (mean ± SD) in comparison to preregimen values. (c) Surface hardness of ostrich eggshell as a percentage of preregimen values (mean ± SD) (100% hardness). (d) Values for ostrich eggshell surface loss in micrometer(mean ± SD) in comparison to preregimen values.

Figure 3a shows a plot of surface hardness change versus preregimen values (100% relative hardness). The plot demonstrates that hardness has remained constant. In general, the percentage values of hardness were very close to 100%. Welch's *t*‐test analysis was performed to determine if the preventive intervention had any influence on the surface hardness loss percentage. No significant effect of protection on surface hardness was observed (*p* > .05). Thus, the protective measure studied, Fluor Protector S, had no statistically significant influence on the measured surface hardness in this regard.

##### Surface loss

Figure 3b shows a plot of mean surface loss relative to preregimen profile values. It demonstrates that, on average, protected specimens had lower surface loss values than unprotected ones. Welch's *t*‐test analysis was performed to determine if the protective measure component had any effect on the quantity of surface loss. On the other hand, a highly significant effect of protection on surface loss was discovered (*p* = .0001). Thus, Fluor Protector greatly reduced enamel surface loss.

#### 3.2.2 Ostrich (*S. camelus*) eggshell

##### Surface hardness

Figure 3c depicts a plot of surface hardness change relative to baseline readings (100% relative hardness). The plot demonstrates that, on average, the protected specimens had much higher surface hardness values than the unprotected specimens. Statistical analysis using Welch's *t*‐test was performed to determine if the preventive action had any influence on the surface hardness. Protection had a highly significant effect on surface hardness (*p* = .0001). Thus, Fluor Protector S greatly reduced the deterioration of the ostrich eggshell's surface hardness.

##### Surface loss

A plot of mean surface loss (Figure 3d) relative to preregimen profile values demonstrates that, on average, protected specimens had significantly lower surface loss values than unprotected ones. Welch's *t*‐test analysis was performed to determine if the preventive treatment had any influence on the quantity of surface loss. Regimen has a highly significant effect on surface loss (*p* = .0001). As a result, Fluor Protector S considerably slowed the rate of surface erosion.

## 4 DISCUSSION

The null hypothesis of this study was partially accepted. In response to different regimens that resembled the consumption of an acidic beverage, the surface loss of ostrich eggshells increased, and the hardness values decreased. Furthermore, as a result of an acidic challenge, ostrich eggshell specimens showed predictable surface loss, hardness drop, and ion loss. However, the effect on both substrates was not entirely similar, because the transient hardness loss phase that was only observed in human enamel, which manifested as an overlooked decrease in surface hardness despite significant ion and structural loss, suggests that human enamel falls short in terms of surface hardness predictability when compared to ostrich eggshells.

For preliminary testing on dental erosion, ostrich eggshell specimens were chosen as substrates. In discussing the findings of this study, it is worthwhile highlighting two advantages of this substrate. To begin with, this substrate is readily available, as it can be purchased on online marketplaces (such as Amazon) for less than US$30 due to its high demand among artists and crafters. Second, the egg is relatively large, which facilitates the preparation of flat samples and the generation of a large number of specimens from a single egg (i.e., more than 500 10 mm^2^ specimens).

Numerous researchers have explored the possibility of using ostrich eggshell to replace bone in reconstructive procedures (Koşarsoy Ağçeli, 2022; Opris et al., 2020). The biocompatibility of ostrich eggshells was also evaluated, as was their capacity to facilitate healing (Durmuş et al., 2008; Kumar et al., 2022). The eggshell's similarity in structure to mineralized bone matrix, along with its manageability and compact size, are all factors in the success of such endeavors. The organic constituents, which account for 2% of the composition of the eggshell, comprise proteins and proteoglycans, which can alter the rate of calcium carbonate crystal precipitation and the form of the crystals. For example, osteoponin stimulates osteoblastic activity and has the ability to bind to hydroxyapatite (Durmuş et al., 2008). Ostrich eggshell, on the other hand, has not yet been used in dental research.

Despite the structural differences between ostrich eggshell and human enamel, surface loss values for single‐dosed regimens were comparable to their human counterparts. Although ostrich eggshell was more prone to demineralization than enamel due to its amorphous crystalline structure (Richards et al., 2002), this structural variation may have contributed to the surface hardness of the substrate appearing to respond more predictably to erosive challenges.

The effects of different regimes on the hardness of ostrich eggshells were greater than on enamel. High‐dose erosive cycles caused predictable and similar trends in hardness loss for both substrates, but lower doses did not. This suggests that the weaker structure of ostrich eggshells may facilitate acid penetration and promote erosion, but it also allows for earlier detection of tooth erosion in preliminary screening studies compared to human enamel, which may not show signs of erosion without surface profilometry. The difference in substrates could be due to a “transient” hardness loss phase in human enamel samples that was not detected, leading to a false indication of acid resistance based solely on hardness loss values. Regimens 1 and 2 showed no or very little hardness loss in enamel, but surface loss occurred, highlighting the need to consider surface loss when interpreting results based on hardness loss.

**Figure 4:**
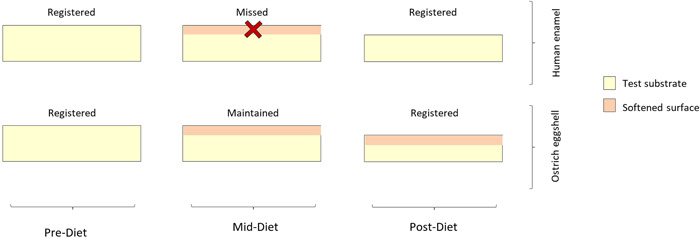
A diagram illustrating the omitted “surface hardness loss” phase when testing human enamel samples for surface hardness following an erosive challenge, which is not the case with ostrich eggshell samples.

In this in vitro study, surface erosion was observed, but none of the postregimen relative hardness percentage values for human enamel was less than 90%, which may not reflect the actual damage caused by the erosive challenge. If surface damage assessment relied solely on hardness testing, false findings could be drawn, as shown in regimens 1 and 2 where no to very minimal surface hardness loss was detected. Figure 4 postulates a brief “hardness loss” phase during regimens that were likely missed by the hardness tester. This has been mentioned previously in the literature (Van Eygen et al., 2005); it was assumed to account for the loss of structure despite the fact that the hardness remained constant, rendering hardness loss during the erosive cycle undetectable. This makes hardness testing problematic for assessing dental erosion in vitro if used solely. However, in ostrich eggshell specimens, this phenomenon was not demonstrated. The observed hardness values for eggshell following the test regimens may accurately reflect the degree of surface tissue loss and provide a more complete picture of the process of tooth erosion than enamel specimens. This could be due to the erosion events taking place in deeper parts of eggshell specimens, which despite losing their structural integrity, still had predicted hardness values that are more indicative of the process of tooth erosion than enamel specimens (Figure 4).

The response of ostrich eggshell specimens to an acidic challenge revealed a predictable and interdependent relationship between hardness and structural integrity. Regardless of the regimen used, the specimens showed both a decrease in hardness and a loss of surface area, indicating that the higher the surface loss, the lower the hardness. This suggests that the transient “hardness loss” phase in eggshell specimens was reliably picked up by the hardness tester.

However, when it comes to in vitro experiments with arbitrary designs that that do not consider the de‐/remineralization cycling, recording the temporary hardness loss is a game of chance. Some in vitro models have shown that relative hardness percentage values can fall within a narrow range of 45%–65% after several days of continuous daily acidic exposure via immersion (Maupomé et al., 1999; Murakami et al., 2009; Passos et al., 2013; Wongkhantee et al., 2006; Xavier et al., 2015). But in studies where specimens were exposed to a test beverage for a shorter time, high relative hardness percentage values of 90%–98% were reported (Panich & Poolthong, 2009; Torres et al., 2010). It is possible that in the studies with lower relative hardness values, the corresponding experiments were able to “register” the transient “hardness loss” phase. In contrast, the transient phase was most likely missed in the studies with higher relative hardness values, despite the prolonged daily acidic exposure.

These limitations of hardness testing in assessing tooth surface loss in enamel highlight the importance of considering other assessment methods in addition to hardness testing. The relative hardness percentage values observed in an in vitro de‐/remineralization cycling model used to test the effect of an erosive drink (regular cola) on enamel were in the range of 83%–86% (Van Eygen et al., 2005). However, the authors noted that these values did not reflect the true value of the hardness drop, but rather the hardness of the newly exposed surface as a result of surface loss due to erosion. This suggests that relying solely on hardness testing in such situations may lead to erroneous findings and hinder an accurate assessment of tooth erosion.

In vitro experiments with unjustifiably prolonged erosive exposure times oversimplify a complex process and are not reflective of real‐world conditions. However, there is a lack of consistent design in dental literature, making comparisons difficult. To address this, researchers should use standardized experimental designs with realistic erosive exposure times and physiological salivary flow rates (Qutieshat et al., 2015). In situ designs now, for instance, tend to immerse the appliance in erosive solutions, which is more ethical than having subjects consume the erosive drink naturally, but does not reflect realistic consumption patterns. The transient “hardness loss” phase and significant enamel “hardening” after each acidic exposure should also be taken into consideration. Several researchers have undertaken in vitro erosion tests to determine the influence of a normal cola drink on the ionic composition of human enamel (Cochrane et al., 2009; Jensdottir et al., 2005; Larsen & Richards, 2002; Willershausen & Schulz‐Dobrick, 2004). Two of these studies (Cochrane et al., 2009; Jensdottir et al., 2005) used an immersion exposure method that is abnormally lengthy and can result in altered surface ion behavior.

According to one study, the rate of calcium ion loss due to erosive exposure was 0.46 mM/h (Larsen & Richards, 2002). Meanwhile, another study found that the rate of calcium ion loss and phosphate ion loss during de‐/remineralization cycling was 0.43 and 0.52 mM/h, respectively (Xavier et al., 2015). Although the calcium‐to‐phosphate ratios in this investigation were similar to those reported in the literature, the overall amount of ion loss was smaller.

Calcium ion loss in enamel after regimens 1 and 2 was between 18.2 and 20.7 mM and phosphate loss was between 25.6 and 30.9 mM, whereas eggshell calcium and phosphate ion loss values were between 20.0–28.4 and 30.0–34.9 mM, respectively. This is consistent with the findings of a study that examined the mineral loss in enamel following exposure to an erosive drink (regular cola) and demonstrated that enamel loses a consistent calcium‐to‐phosphate ratio during the erosive process (Willershausen & Schulz‐Dobrick, 2004).

Once again, this study found a dose–response relationship, with single‐dosed regimens losing much fewer ions than double‐dosed regimens. As demonstrated by the aforementioned comparisons, all surface effects tested (surface hardness, surface loss, and ion loss) show predictable behavior in eggshell, whereas enamel specimens exhibit predictable behavior for surface loss and ion loss, but fall short when it comes to surface hardness, where, despite the presence of a dose–response relationship, the loss of surface hardness in single‐dosed regimens is missed.

The predictable ostrich eggshell behavior reported in this work can be explained by the fact that the presence of phosphate increases the solubility of calcium carbonate, the main component of Ostrich eggshell, and the presence of bicarbonate increases the solubility of calcium phosphate in acidic environments (Greenwald, 1945). It is worth mentioning that the standard test drink utilized in this research (regular cola) includes carbonic and phosphoric acid and has a pH of 2.47.

This study found that a fluoride varnish used as a preventive treatment was effective in reducing tooth erosion. This is consistent with a number of studies demonstrating the preventive impact of fluoride varnishes (Bezerra et al., 2021; Moharramkhani et al., 2021). The varnish significantly lowered the rate of surface loss in both human enamel and ostrich eggshell specimens. Previous in vitro and in situ studies have also demonstrated the preventative impact of fluoride varnishes (Magalhães et al., 2008; Vieira et al., 2007). In terms of hardness, a study found that human enamel treated with fluoride varnish experienced less surface hardness loss than the control group (Murakami et al., 2009), and a similar finding was observed in ostrich eggshell specimens treated with fluoride varnish. However, no hardness loss was observed in the treated human enamel specimens, and the treated group did not exhibit any hardness loss either. The authors suggest that this may be due to the natural remineralization process and buffering capacity of saliva, which make the enamel surface more resistant to mild or brief erosive challenges in real‐life scenarios.

The preventive treatment utilized in this study exhibited favorable effects against tooth erosion. Specifically, the fluoride varnish demonstrated the ability to significantly decrease the rate of surface loss in both human enamel and ostrich eggshell, from 10.09 to 1.82 µm/h and 5.98 to 1.04 µm/h, respectively (Table 2). These results are consistent with previous studies that have also demonstrated the preventive impact of fluoride varnishes (Bezerra et al., 2021; Moharramkhani et al., 2021).

**Table 2:** Rate (mean ± SD) of surface loss reported in the “validation” and “application” sections of this work to allow for comparison with other works reported in the literature.

Surface loss rate	Regimen 1	Regimen 2	Regimen 3	Regimen 4
Validation				
Human enamel (*N* = 16) (μm/h)	10.09 ± 2.31	8.78 ± 2.39	10.49 ± 2.22	8.44 ± 1.53
Ostrich eggshell (*N* = 16)	5.90 ± 2.37	6.96 ± 2.51	7.43 ± 1.92	6.09 ± 1.74

Surface loss rate	Unprotected	Protected
Application		
Human enamel (*N* = 16) (μm/h)	10.09 ± 2.31	1.82 ± 0.60
Ostrich eggshell (*N* = 28)	5.98 ± 2.35	1.04 ± 0.41

In an in vitro investigation that evaluated the preventive effect of a fluoride varnish on erosion, the rate of surface loss in bovine enamel was significantly reduced from 9.16 to 1.73 µm/h after 3 days of 6‐min per day exposure to an erosive drink (Magalhães et al., 2008). Furthermore, an in situ examination of human enamel specimens found that the rate of surface degradation decreased from 10 to 14 to 1–5 µm/h following the application of a fluoride varnish (Vieira et al., 2007). These findings support the beneficial preventative effects of the fluoride varnish described in this study.

Regarding hardness, a study using demineralization and remineralization cycling in vitro found that the fluoride varnish was effective in preserving the surface hardness of human enamel, with the control group exhibiting a greater loss of surface hardness than the fluoride varnish group (56.9% vs. 72.6%, respectively) (Murakami et al., 2009). A similar trend was observed in the ostrich eggshell specimens treated with fluoride varnish, as these specimens maintained 91.59% of their relative hardness compared to 69.03% in the control group. However, no loss of hardness was observed in the treated group of human enamel specimens, which may be attributed to the natural remineralization process and buffering capacity of saliva. The authors suggest that the “realistic” experimental setting utilized in this study may have contributed to this outcome, as it more closely mimics real‐life scenarios (Qutieshat et al., 2015, 2018). Overall, the findings of this study add to the growing body of evidence supporting the effectiveness of fluoride varnishes in preventing tooth erosion and preserving surface hardness.

While the current study was conducted under strict control, particularly after adopting actual human drinking behavior values (Qutieshat et al., 2015), one may argue that the amount of tissue loss was subject to structural and biological variation within the same substrate specimens (i.e., variability in the source, location, and history). This is unlikely to have occurred, considering that standard deviation values between enamel and eggshell specimens were not greater than 15% in either of the substrates (13.2% and 12.5%, respectively, based on a total of 728 specimens tested). Hardness differences of up to 16% have previously been recorded in the literature for human enamel (Turssi et al., 2010). Additionally, the fact that all of the eggshell samples used in this experiment came from the same egg helped to reduce the amount of uncertainty that might have been caused by the presence of biological and structural variations.

One limitation of the current study is that abrasive forces were not considered. Because erosion and abrasion are inseparably linked, they must both be considered in any analysis of tooth surface loss. However, because we did not use any mechanical means in our research, abrasion was absent, and we concentrated solely on erosion.

## 5 CONCLUSION

This study partially accepted the null hypothesis as the effect of different acidic regimens on ostrich eggshells and human enamel was not entirely similar. Ostrich eggshell specimens proved to be a suitable substrate for preliminary testing on dental erosion, as they are readily available and can generate a large number of specimens from a single egg. The study also highlighted the potential of ostrich eggshells for dental research as they respond more predictably to erosive challenges, and the difference in substrates suggests that the weaker structure of ostrich eggshells may facilitate acid penetration and promote erosion, but it also allows for earlier detection of tooth erosion in preliminary screening studies compared to human enamel. The findings also emphasize the need to consider surface loss when interpreting results based on hardness loss, as hardness testing alone may provide false findings. The divergent response of human enamel and ostrich eggshell to erosion in the presence of artificial saliva is likely due to variations in their inherent structure, chemical composition, and biological interaction. Overall, the study suggests that ostrich eggshell could be a viable alternative to human teeth in assessing dental erosion, and the findings highlight the importance of recognizing the differences between substrates in evaluating tissue recovery.

## AUTHOR CONTRIBUTIONS

In this study, which is part of a postdoctoral research project, the following contributions were made by the authors: Abubaker Qutieshat contributed to conceptualization, data curation, methodology, investigation, formal analysis, writing—original draft preparation, and visualization. Richard Graham Chadwick contributed to conceptualization, data curation, resources, validation, supervision, and writing—review and editing. Andrew Graham Mason contributed to data curation, project administration, supervision, and writing—review and editing.

## CONFLICT OF INTEREST STATEMENT

The authors declare no conflict of interest.

## Data Availability

The data that support the findings of this study are available from the corresponding author upon reasonable request.
